# Supplementary material for: Empirical methods for controlling false positives and estimating confidence in ChIP-Seq peaks
Source: BMC Bioinformatics. 2008 Dec 5;9:523. doi: 10.1186/1471-2105-9-523 (PMC2628906; doi:10.1186/1471-2105-9-523)
Supplement: Additional File 1 — A variety of html documents from the USeq web site detailing the available applications, their best usage, output file type descriptions, command line menus, etc. [file 1471-2105-9-523-S1.zip › USeqUserGuides/JavaDoc.zip!/JavaDoc/edu/utah/seq/data/Info.html]

Info


|  |  |  |
| --- | --- | --- |
| **Overview** \| **Package** \| **Class** \| **Use** \| **Tree** \| **Deprecated** \| **Index** \| **Help** | |  |
| **PREV CLASS**   **NEXT CLASS** | **FRAMES**    **NO FRAMES**     **All Classes** |  |
| SUMMARY: NESTED \| FIELD \| CONSTR \| METHOD | DETAIL: FIELD \| CONSTR \| METHOD |  |


---


## edu.utah.seq.data Class Info

```
java.lang.Object
  edu.utah.seq.data.Info
```

**All Implemented Interfaces:**: java.io.Serializable

---

``` public class Info extends java.lang.Object implements java.io.Serializable ```

Data file information.

**See Also:**: Serialized Form

---

| **Field Summary** | |
| --- | --- |
| `static long` | `serialVersionUID` |


**Constructor Summary**

`Info(java.lang.String name,
java.lang.String versionedGenome,
java.lang.String chromosome,
java.lang.String strand,
int readLength,
java.util.HashMap<java.lang.String,java.lang.String> notes)`
  
          Notes, can be null, everything else is required.


| **Method Summary** | |
| --- | --- |
| `java.lang.String` | `getChromosome()` |
| `java.lang.String` | `getName()` |
| `java.util.HashMap<java.lang.String,java.lang.String>` | `getNotes()` |
| `int` | `getReadLength()` |
| `java.lang.String` | `getStrand()` |
| `java.lang.String` | `getVersionedGenome()` |
| `void` | `setChromosome(java.lang.String chromosome)` |
| `void` | `setName(java.lang.String name)` |
| `void` | `setNotes(java.util.HashMap<java.lang.String,java.lang.String> notes)` |
| `void` | `setReadLength(int readLength)` |
| `void` | `setStrand(java.lang.String strand)` |
| `void` | `setVersionedGenome(java.lang.String versionedGenome)` |
| `java.lang.String` | `toString()` |

**Methods inherited from class java.lang.Object**

`equals, getClass, hashCode, notify, notifyAll, wait, wait, wait`

**Field Detail**

### serialVersionUID

```
public static final long serialVersionUID
```

**See Also:**: Constant Field Values


**Constructor Detail**

### Info

```
public Info(java.lang.String name,
            java.lang.String versionedGenome,
            java.lang.String chromosome,
            java.lang.String strand,
            int readLength,
            java.util.HashMap<java.lang.String,java.lang.String> notes)
```

:   Notes, can be null, everything else is required.

    **Parameters:**: `versionedGenome` - - As defined by UCSC (ie hg18, dm2, ce2, mm8), see http://genome.ucsc.edu/FAQ/FAQreleases: `chromosome` - - As defined by UCSC see http://genome.ucsc.edu/ and particular species.: `strand` - - +, -, or .: `readLength` - - Length of the individual reads.: `notes` - - optional can be null.


**Method Detail**

### toString

```
public java.lang.String toString()
```

:   **Overrides:**: `toString` in class `java.lang.Object`

---


### getChromosome

```
public java.lang.String getChromosome()
```

---


### setChromosome

```
public void setChromosome(java.lang.String chromosome)
```

---


### getName

```
public java.lang.String getName()
```

---


### setName

```
public void setName(java.lang.String name)
```

---


### getNotes

```
public java.util.HashMap<java.lang.String,java.lang.String> getNotes()
```

---


### setNotes

```
public void setNotes(java.util.HashMap<java.lang.String,java.lang.String> notes)
```

---


### getStrand

```
public java.lang.String getStrand()
```

---


### setStrand

```
public void setStrand(java.lang.String strand)
```

---


### getVersionedGenome

```
public java.lang.String getVersionedGenome()
```

---


### setVersionedGenome

```
public void setVersionedGenome(java.lang.String versionedGenome)
```

---


### getReadLength

```
public int getReadLength()
```

---


### setReadLength

```
public void setReadLength(int readLength)
```


---


|  |  |  |
| --- | --- | --- |
| **Overview** \| **Package** \| **Class** \| **Use** \| **Tree** \| **Deprecated** \| **Index** \| **Help** | |  |
| **PREV CLASS**   **NEXT CLASS** | **FRAMES**    **NO FRAMES**     **All Classes** |  |
| SUMMARY: NESTED \| FIELD \| CONSTR \| METHOD | DETAIL: FIELD \| CONSTR \| METHOD |  |


---
